# B7-H4 Inhibits the Development of Primary Sjögren's Syndrome by Regulating Treg Differentiation in NOD/Ltj Mice

**DOI:** 10.1155/2020/4896727

**Published:** 2020-09-27

**Authors:** Xu Zheng, Qikai Wang, Xiang Yuan, Yingbo Zhou, Hui Chu, Guosheng Wang, Xiangpei Li, Yiping Wang, Li Wei, Li Wang, Xiaomei Li

**Affiliations:** ^1^Department of Rheumatology and Immunology, The First Affiliated Hospital of USTC, University of Science and Technology of China, Hefei, Anhui, China; ^2^Department of Rheumatology and Immunology, The People's Hospital of Bozhou, Bozhou, Anhui, China; ^3^Westmead Institute for Medical Research, University of Sydney, Westmead 2145 NSW, Australia; ^4^Pharmacoepidemiology and Medication Safety Research Cluster, UCL School of Pharmacy, London WC1N 1AX, UK

## Abstract

**Background:**

This study is aimed at exploring the role of B7-H4 in the pathogenesis of primary Sjögren's syndrome (pSS) in NOD/Ltj mouse.

**Methods:**

B7-H4 expression in salivary glands was examined by IHC, and lymphocyte infiltration was showed by H&E. Next, anti-B7-H4 mAb or immunoglobulin isotype was injected into NOD/Ltj mice. Cytokine levels were measured by quantitative RT-PCR, and immunoglobulins were measured by ELISA. T cell subsets were analyzed by flow cytometry. Last, we treated NOD/Ltj mice with B7-H4Ig and control Ig. CD4+Foxp3+ T cells were assessed by immunohistochemistry. Two-tailed Student's *t*-tests were used to detect the statistical difference in various measures between the two groups.

**Results:**

B7-H4 expression was remarkably reduced in salivary glands of NOD/Ltj mice at 15 weeks compared with the NOD/Ltj mice at 8 weeks. Anti-B7-H4 mAb treatment increased lymphocyte infiltration in salivary glands. Inflammatory cytokines including IL-12, IL-18, IL-1*α*, TNF-*α*, IFN-*α*, and BAFF were upregulated markedly in anti-B7-H4 mAb-treated mice compared to IgG isotype-treated mice. Flow cytometry analysis showed that anti-B7-H4 mAb-treated mice had lower levels of CD4+Foxp3+/CD4+ T cells in spleen. Moreover, Foxp3 mRNA levels of salivary glands were diminished in anti-B7-H4 mAb-treated mice. Flow cytometry analysis showed that anti-B7-H4 mAb inhibited CD4+Foxp3+/CD4+ T cell production, while B7-H4Ig would promote naïve CD4+ T into Treg differentiation. Administration with B7-H4Ig displayed significantly decreased lymphocyte infiltration in salivary glands and low levels of total IgM and IgG in serum. Analysis of inflammatory cytokines in salivary glands after B7-H4Ig treatment revealed that the mRNA levels of IL-12, IL-6, IL-18, IL-1*α*, TNF-*α*, and IFN-*α* were significantly downregulated in B7-H4Ig-treated mice compared to control Ig treatment. B7-H4Ig-treated mice had significantly higher levels of CD4+Foxp3+/CD4+ T cells in spleen. IHC in salivary gland revealed that CD4+Foxp3+ T cells of B7-H4Ig treatment mouse were more than control Ig treatment.

**Conclusions:**

Our findings implicate that B7-H4 has a protective role for salivary gland epithelial cells (SGECs) and therapeutic potential in the treatment of pSS.

## 1. Introduction

Primary Sjögren's syndrome is a chronic, inflammatory autoimmune disease characterized by lymphocytic infiltration in the exocrine glands, especially the salivary and lacrimal glands, leading to a destruction of their functional components [1]. The disease may occur as primary Sjögren's syndrome (pSS) alone or in conjunction with another autoimmune disorder as secondary Sjögren's syndrome [2]. Although dry mouth and eyes are the hallmark symptoms of pSS, the disease affects other organs of the body and causes substantial morbidity [3]. Up to now, the underlying pathophysiologic mechanisms of Sjögren's syndrome remain obscure. NOD/Ltj is the most widely used pSS model animal exhibiting CD4+ lymphocyte infiltration, autoantibodies, and xerostomia. Analysis of lesion tissue of the salivary glands shows a predominance of T lymphocytes surrounding ductal epithelial cells. The 70%~80% of these T cells are CD4+ T cells, 10% are CD8+ T cells, and the remaining infiltrating cells are B cells [4, 5]. In recent years, evidences have indicated that salivary gland epithelial cells (SGECs) in SS lesions were activated and played an important role in the induction and perpetuation of the inflammatory processes [6, 7]. Presence of costimulatory factors CD80, CD86, and CD40 on SGEC is capable to activate immune cells to secret Th1 cytokines. This leads a feed forward loop, resulting in upregulating expression of costimulatory molecules and adhesion molecules on SGEC [7].

B7-H4 is a member of the B7/CD28 costimulatory/coinhibitory superfamily. The role of B7-H4 in the inhibition of immune responses has shown in various in vitro [8–11] and in vivo studies [12–15]. Studies in experimental autoimmune encephalomyelitis (EAE) model have shown that blockage of endogenous B7-H4 by specific monoclonal antibody can promote T cell responses and accelerate the disease. These results indicate that B7-H4 has a capacity to regulate autoreactive T cell responses [9]. Moreover, deficiency in expression of B7-H4 in SGECs from pSS patients causes the lack of suppression of infiltrating CD4+ T cells [16].

In this study, we explored the regulatory mechanism of B7-H4 in the pathogenesis of pSS through B7-H4 monoclonal antibody or soluble B7-H4Ig fusion protein (intervening an Fc fusion of the extracellular domain of B7-H4 to mimic the natural ability of B7-H4). Functionally, anti-B7-H4 mAb-induced aggravation of pSS correlates with the decreased numbers and functions of Tregs in vivo and in vitro. The present results show that B7-H4 is expressed in glandular epithelial cells [16], and blockade of B7-H4 increases proinflammatory cytokines such as IL-12, IL-18, IL-1*α*, TNF-*α*, IFN-*α*, and BAFF, while reduces numbers of Tregs in NOD/Ltj mouse. Moreover, in salivary gland, we find that both TGF-*β* and IL-2 were reduced by anti-B7-H4 mAb [17]. In this study, we also found that B7-H4Ig treatment in NOD/Ltj mouse reduces proinflammatory cytokines, ameliorates lymphocyte infiltration, and increases the number and function of Tregs.

## 2. Methods

### 2.1. Mice Strains

Female nonobese diabetic (NOD/Ltj) mice or C57BL/6 (B6) mice were used in aged 8 weeks and were housed in a specific pathogen-free (SPF) room within the mouse facility of the Central Laboratory at Anhui Provincial Hospital. Mouse housing procedures were conducted according to the Guide for the Care and Use of Medical Laboratory Animals (Ministry of Health, China, 1998), and the breeding and use of these animals for the present studies were approved by the Institutional Animal Care and Use Committee of Anhui Provincial Hospital.

### 2.2. In Vivo Treatments

8 weeks of NOD/Ltj mice were given i.p. injections with different doses of recombinant anti-B7-H4 mAb (AMP-110, Amplimmune) or IgG isotype (BD Bioscience). NOD/Ltj mice received 12.5 mg/kg of anti-B7-H4 mAb or the same dosage of IgG isotype for the first time, then were administered with anti-B7-H4 mAb 7.5 mg/kg or IgG isotype 7.5 mg/kg every 3 days for 2 weeks. To determine the therapeutic effect of B7-H4Ig (Chimerigen laboratories), female NOD/Ltj mice were administered with B7-H4Ig 7.5 mg/kg or control Ig (Chimerigen laboratories) 7.5 mg/kg every 3 days for 2 weeks [15] starting at the age of 8 weeks. All mice were sacrificed at 15 weeks of age.

### 2.3. Flow Cytometry Analysis and Antibodies

Percp-5.5 anti-mouse CD4, FITC anti-mouse CD3, APC anti-mouse IFN-*γ*, APC anti-mouse Foxp3 and PE anti-mouse IL17*α*, and isotype-matched mAbs were purchased from BD Biosciences; Fixation/Permeabilization Kit was purchased from eBioscience. Single-cell suspensions were generated from spleens and treated with RBC lysis buffer containing NH4Cl. Cells from spleen were fluorescently labeled by incubation with the indicated Abs in FACS buffer (PBS containing 0.3% BSA) for 30 min on ice. Subsequently, samples were washed and suspended in PBS containing 1% FCS. For intracellular cytokine analysis of IL-17A and IFN-*γ*, the splenocytes were stimulated for 4 h at 37°C with PMA (50 ng/ml; Sigma) and ionomycin (1 *μ*g/ml; Sigma). Then, cells were permeabilized and fixed after surface staining and stained with fluorescence-conjugated antibodies or IgG controls. Data were acquired on a BD FACS CantoII.

### 2.4. Treg Induction Experiment

Naïve CD4+ T cells were isolated from the splenocytes using naive CD4+ T Cell Isolation Kit (Miltenyi), 5 × 10^5 cells/well were cultured with SGECs (5 × 10^5 cells/well) and stimulated with 1 *μ*g/ml anti-CD3 (eBioscience) and 2 *μ*g/ml anti-CD28 (eBioscience) plus anti-B7-H4 mAb (10 *μ*g/ml) or IgG isotype (10 *μ*g/ml) or B7-H4Ig (10 *μ*g/ml) or control Ig (10 *μ*g/ml). Cells were cultured in Treg-promoting (IL-2 30-50 U/ml, PeproTech, TGF-*β* 2 ng/ml, R&D Systems) conditions. After 3 days of culture, the percentage of Treg cells was detected by flow cytometry.

### 2.5. Pathological Assessment

NOD/Ltj mice were killed at 15 weeks of age, and blood serum had been collected. Submandibular salivary glands were snap frozen in OCT compound and cut into 6-*μ*m serial sections (Zeiss); for histochemistry, salivary glands were embedded in paraffin, then sectioned (5 *μ*m each) and stained with H&E. The focus score (FS) was defined as an aggregate of 50 or more lymphocytes per 4 mm^2^ of salivary gland tissue and stained with H&E for histological observation of mononuclear cell infiltration. Histological observations and photomicrography were performed using an Olympus microscope. For immunohistochemistry analysis, immunohistochemistry for mouse Foxp3 was performed as previously described, with minor modifications [18]. The antibodies used in this study were as follows: PE anti-mouse CD4 (Abcam), FITC anti-mouse Foxp3 (Abcam), and diaminidophenylindol (DAPI, Invitrogen). The number of CD4+ and CD4+Foxp3+ was counted in 5 consecutive high-power fields (40x) by means of 0.0314 mm^2^ graticule fitted in the eyepiece of the microscope and expressed as ratio of CD4+Fopx3+/CD4+ T cells.

### 2.6. Quantitative Real-Time PCR (qRT-PCR) Assay

Total RNA was extracted using RNeasy (Qiagen) and was reverse transcribed to cDNA using Taqman reverse transcription reagents (Applied Biosystems) according to the manufacturer's instructions. Primer and probe sets were obtained from Applied Biosystems. qRT-PCR was performed using the Taqman Universal PCR Master Mix. Primers used to amplify specific gene fragments as follows:

IL-1*α*: 5′AAGACAAGCCTGTGTTGCTGAAGG (forward) and 5′TCCCAGAAGAAAATGAGGTCGGTC (reverse); TNF-*α*: 5′AGAAGTTCCCAAATGGCCT (forward) and 5′CCACTTGGTGGTTTGCTACG (reverse); IL-10: 5′CCAAGCCTTATCGGAAATGA (forward) and 5′TTTTCACAGGGGAGAAATCG (reverse); IL-2: 5′TGAGCAGGATGGAGAATTACAGG (forward) and 5′GTCCAAGTTCATCTTCTAGGCAC (reverse); TGF-*β*: 5′GGAAATCAACGGGATCAGCC (forward) and 5′GTGCCGTGAGCTGTGCAGGT (reverse); Foxp3: 5′CGAAAGTGGCAGAGAGGTATT (forward) and 5′GCATGGGTCTGTCTTCTCTAAG (reverse); IL-18: 5′CTCTGTGGTTCCATGCTTTCT (forward) and 5′GTTTGAGGCGGCTTTCTTTG (reverse); IL-6: 5′TGAACTCCTTCTCCACAAGCG (forward) and 5′TCTGAAGAGGTGAGTGGCTGTC (reverse); IL-12: 5′TCAAACCAGACCCACCGAA (forward) and 5′GCTGACCTCCACCTGCTGA (reverse); IFN-*α*: 5′GGCTCTGGTGCATGAGATGT (forward) and 5′GCCTTCTTCCTGAATCTGTCTTA (reverse); BAFF: 5′AAGACCTACGCCATGGGACATC(forward) and 5′TCTTGGTATTGCAAGTTGGAGTTCA (reverse); *β*-actin: 5′TACAGCTTCACCACCACAGC (forward) and 5′TCTCCAGGGAGGAAGAT (reverse).

### 2.7. Immunoglobulins Measurement

Serum IgM, IgG, and IgA were detected using ELISA kits. 96-well plates were coated with 50–100 *μ*L capture antibodies for overnight at 4°C or 2 h in room temperature following the manufacturer's instruction. Wash wells four times in using PBST and add 100 *μ*L diluted serum or standard incubating at room temperature for 2 h. Wash wells four times and add 100 *μ*L of diluted detection antibody to each well incubating for 1 h. After washing, add 100 *μ*L of diluted HRP conjugate to each well for 30 min thoroughly aspirate and add 100 *μ*L of substrate to each well and develop plate at room temperature in the dark for 30 min. Stop the reaction by adding 100 *μ*L of stop solution. While the levels of Ig isotypes were read at 405 nm, the levels of autoantibodies were read at 450 nm. The antibody concentrations were calculated using Ig standards, provided by the manufacturer.

### 2.8. Statistical Analysis

The data are expressed as mean ± standard deviation (SD). Two-tailed Student's *t*-tests were used to detect the statistical difference in various measures between the two groups. Data were shown as a representative experiment of three independent experiments. Statistical significance was defined as ^∗^*P* < 0.05, ^∗∗^*P* < 0.01, and ^∗∗∗^*P* < 0.001.

## 3. Results

### 3.1. B7-H4 Suppresses the Progression of pSS in NOD/Ltj Mice

**Figure 1:**
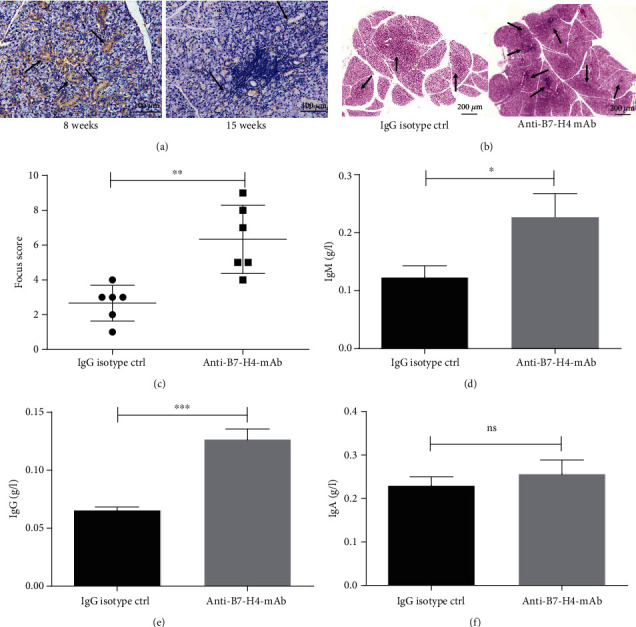
B7-H4 suppresses the progression of pSS in NOD/Ltj mice. (a) B7-H4 expression in salivary glands from 8 weeks or 15 weeks of NOD/Ltj mouse was examined by IHC (100x). Arrows indicate B7-H4 expression. (b, c) Female NOD/Ltj mice were injected with IgG isotype ctrl or anti-B7-H4 mAb from 8 weeks of age. The mice were sacrificed at 15 weeks of age. H&E stains of the salivary gland sections from IgG isotype ctrl treatment and anti-B7-H4 mAb treatment mice. Arrows indicate infiltrates within the salivary gland (b). Histopathological assessment data are presented as FS (c). (d–f) Detection of pSS-associated autoantibodies in serum. Serum samples were collected from IgG isotype ctrl treatment and anti-B7-H4 mAb treatment NOD/Ltj mice, and the levels of immunoglobulins were measured by ELISA. Immunoglobulin levels for (d) IgM, (e) IgG, and (f) IgA are shown. Two-tailed Student's *t*-test. All the data presented were from three independent experiments.

Immunohistochemical analysis of B7-H4 expression in salivary glands revealed that B7-H4 expression was remarkably reduced in salivary glands of NOD/Ltj mice at 15 weeks compared with the NOD/Ltj mice at 8 weeks (Figure 1(a)). Blocking endogenous B7-H4 by injection of anti-B7-H4 mAb, NOD/Ltj mice received 12.5 mg/kg of anti-B7-H4 mAb or the same dosage of IgG isotype for the first time, then were administered with anti-B7-H4 mAb 7.5 mg/kg or IgG isotype 7.5 mg/kg every 3 days for 2 weeks. Salivary gland H&E staining showed aggravated lymphocyte infiltration in salivary glands of anti-B7-H4 mAb treatment when compared with IgG isotype ctrl treatment in NOD/Ltj mice (Figures 1(b) and 1(c)). To evaluate the immune response following anti-B7-H4 mAb treatment, we analyzed the levels of total IgM, IgG, and IgA in the serum of anti-B7-H4 mAb-treated mice and IgG isotype-treated mice. Anti-B7-H4 mAb-treated NOD/Ltj mice displayed elevated levels of total IgG and IgM compared to IgG isotype-treated mice (Figures 1(d) and 1(e)). However, level of IgA did not show any significant differences between the groups (Figure 1(f)).

### 3.2. Blockade of B7-H4 Increases Proinflammatory Cytokines in NOD/Ltj Mice

**Figure 2:**
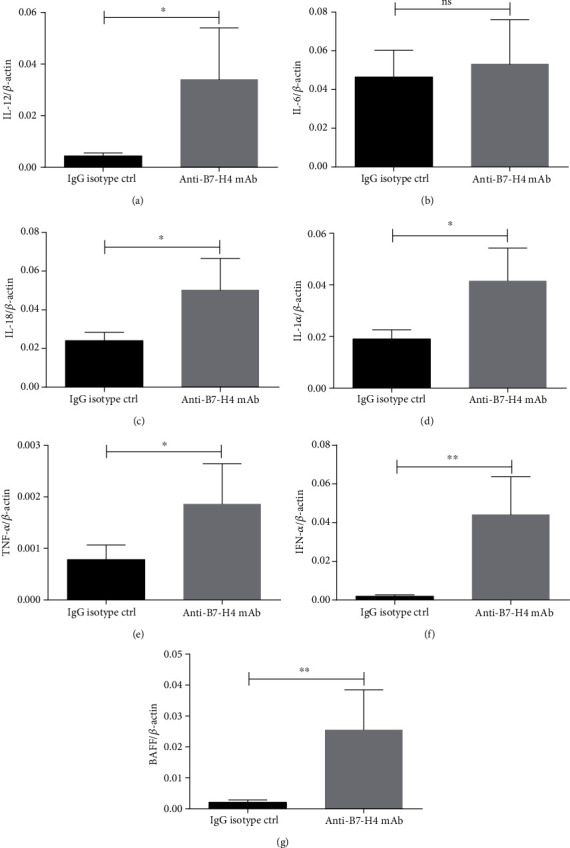
Blockade of B7-H4 increases proinflammatory cytokines in NOD/Ltj mice. (a–g) The salivary glands were collected from IgG isotype ctrl treatment and anti-B7-H4 mAb treatment NOD/Ltj mice. Relative levels of (a) IL-12 mRNA, (b) IL-6 mRNA, (c) IL-18 mRNA, (d) IL-1*α* mRNA, (e) TNF-*α* mRNA, (f) IFN-*α* mRNA, and (g) BAFF mRNA were determined by quantitative RT-PCR. Two-tailed Student's *t*-test. All the data presented were from three independent experiments.

Inflammatory cytokines are induced in pSS [19]. Therefore, we analyzed the levels of inflammatory cytokine mRNA in salivary glands, which were in the relevant to this model. We found that levels of IL-12 (Figure 2(a)), IL-18 (Figure 2(c)), IL-1*α* (Figure 2(d)), TNF-*α* (Figure 2(e)), IFN-*α* (Figure 2(f)), and BAFF (Figure 2(g)) were upregulated markedly in anti-B7-H4 mAb-treated mice compared to IgG isotype-treated mice, while the level of IL-6 in salivary glands showed no difference between the two treatments (Figure 2(b)).

### 3.3. B7-H4 Regulates CD4+ T Cell Responses In Vivo

**Figure 3:**
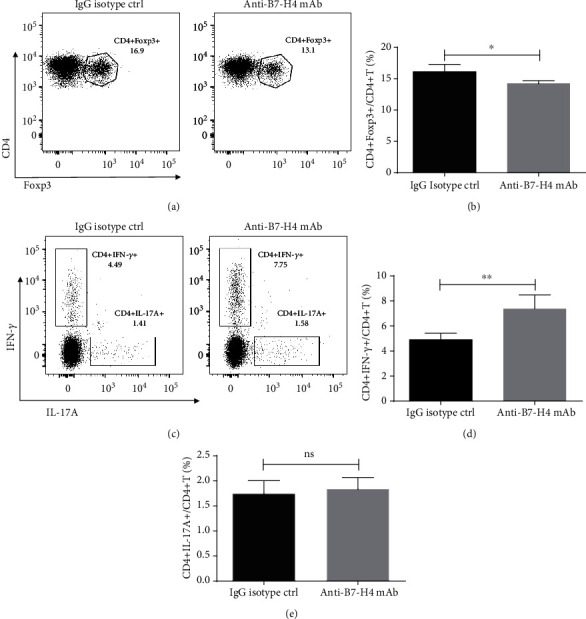
B7-H4 regulates CD4+ T cell response in vivo. (a, b) Splenocytes from 15-week-old NOD/Ltj mice treated with IgG isotype ctrl and anti-B7-H4 mAb, respectively, were stained with anti-CD4/anti-intracellular Foxp3 antibodies and analyzed by flow cytometry (a), and accumulated data of CD4+Foxp3+/CD4+ T cells are shown (b). (c–e) Splenocytes from 15-week-old IgG isotype ctrl or anti-B7-H4 mAb treatment NOD/Ltj mice were stimulated with PMA and ionomycin for 4 h and were stained with anti-CD4/intracellular IFN-*γ* or anti-CD4/intracellular IL-17A antibodies and analyzed by flow cytometry (c), accumulated data of CD4+IFN-*γ*+/CD4+ T cells (d), and CD4+IL-17A+/CD4+ T cells (e). Two-tailed Student's *t*-test. All the data presented were from three independent experiments.

Expression of cytokine mRNA in salivary glands suggests a role of B7-H4 in regulation of T cell immune responses. Flow cytometry analysis of CD4+ T subpopulations in the spleen showed that anti-B7-H4 mAb treatment promoted a relative expansion of the CD4+Foxp3+ T subset (Figures 3(a) and 3(b)) and CD4+IFN-*γ*+ T subset (Figures 3(c) and 3(d)) compared to IgG isotype. In addition, CD4+IL-17A+ T subset (Figures 3(c) and 3(e)) was found at similar levels in the two treatment groups.

### 3.4. Blockade of B7-H4 Suppresses Tregs in Salivary Gland

**Figure 4:**
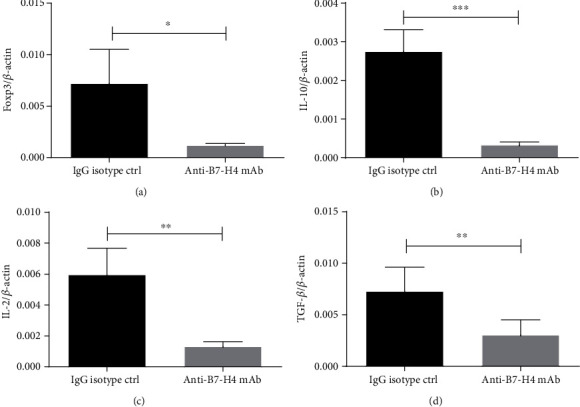
Blockade of B7-H4 suppresses Tregs in salivary gland. (a–d) The salivary glands were collected from IgG isotype ctrl treatment and anti-B7-H4 mAb treatment NOD/Ltj mice. Relative levels of (a) Foxp3 mRNA, (b) IL-10 mRNA, (c) IL-2 mRNA, and (d) TGF-*β* mRNA were determined by quantitative RT-PCR. Two-tailed Student's *t*-test. All the data presented were from three independent experiments.

In salivary glands, Tregs in anti-B7-H4 mAb-treated mice were examined compared to control mice. Foxp3 mRNA levels of salivary glands examined with quantitative RT-PCR were diminished in anti-B7-H4 mAb-treated mice compared to that in IgG isotype-treated mice (Figure 4(a)). Moreover, mRNA level of IL-10, which Tregs functionally dependent upon [20, 21], were remarkably diminished in salivary gland of anti-B7-H4 mAb-treated mice (Figure 4(b)). TGF-*β* in combination with IL-2 is critical for the differentiation of Tregs [22]. IL-2 and TGF-*β* mRNA levels markedly decreased in NOD/Ltj mice injected with anti-B7-H4 mAb as determined by qRT-PCR analysis of salivary glands (Figures 4(c) and 4(d)).

### 3.5. B7-H4 on SGEC Is Involved in Treg Differentiation

**Figure 5:**
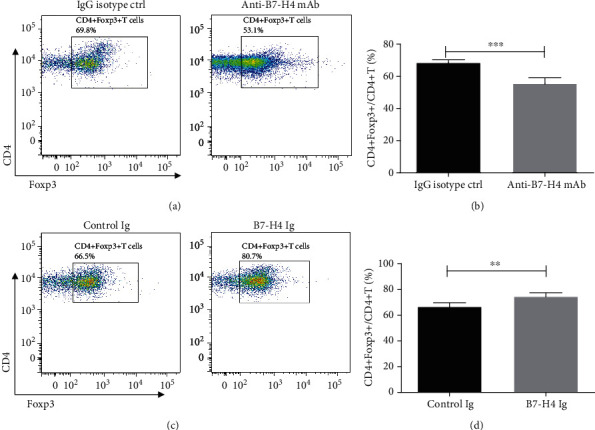
B7-H4 on SGEC is involved in Treg differentiation. (a, b) Naïve CD4+ T cells were isolated from NOD/Ltj mice and cultured (2.5 × 10^5^ cells/well) with anti − CD3 (1 *μ*g/ml) + anti − CD28 (2 *μ*g/ml) plus an equal number of SGECs in the presence of IL-2 (30-50 U/ml), TGF-*β* (2 ng/ml), plus IgG isotype, or anti-B7-H4 mAb. The cells were collected on day 3 of culture, and the percentage of CD4+FoxP3+/CD4+ T cells was determined by FACS analysis (a). Accumulated data of Tregs differentiation in vitro was shown (b). (c, d) Naïve CD4+ T cells from NOD/Ltj mice were activated in Treg-promoting conditions as detailed in (a, b) with control Ig or B7-H4 Ig, and the percentage of CD4+FoxP3+/CD4+ T cells was determined by FACS analysis (c). Accumulated data of Treg differentiation in vitro was shown (d). Two-tailed Student's *t*-test. All the data presented were from three independent experiments.

We next determined whether SGEC B7-H4 is involved in the Treg cell differentiation. The ability of B7-H4 on SGEC under Treg-driving conditions to modulate the differentiation of naïve CD4+ T cells was examined in either containing anti-B7-H4 mAb or IgG isotype. Flow cytometry analysis showed that anti-B7-H4 mAb inhibited CD4+Foxp3+/CD4+ T cell production compared to the addition of IgG isotype (Figures 5(a) and 5(b)). We next asked if B7-H4Ig would promote naïve CD4+ T into Treg differentiation. Naïve CD4+ T was cocultured with SGECs in Treg-promoting conditions with or without B7-H4Ig. The data showed that B7-H4Ig treatment increased CD4+Foxp3+/CD4+ T cells compared with control Ig treatment (Figures 5(c) and 5(d)).

### 3.6. B7-H4Ig Ameliorates pSS in NOD/Ltj Mouse

**Figure 6:**
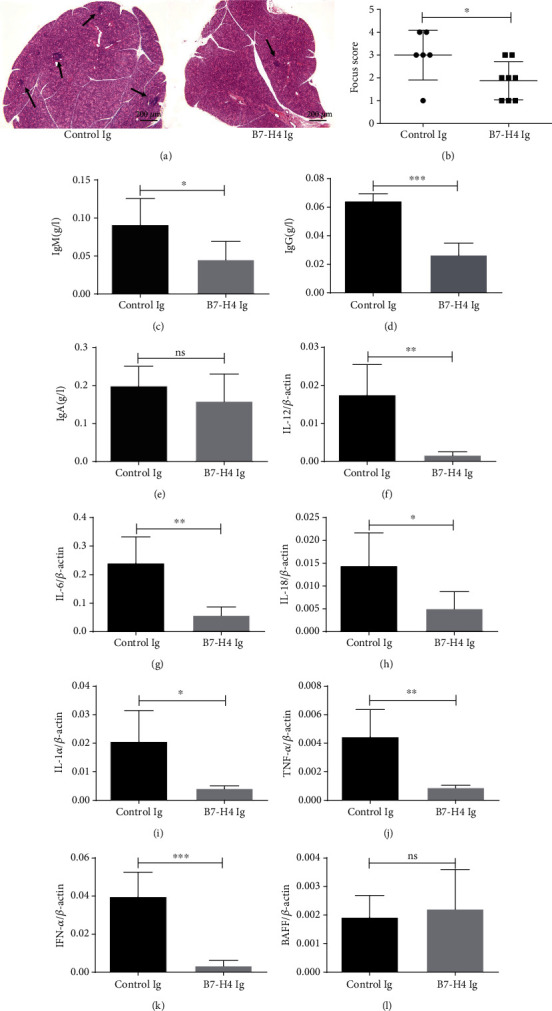
B7-H4Ig ameliorates pSS in NOD/Ltj mouse. (a, b) Female NOD/Ltj mice were injected with control Ig or B7-H4Ig from 8 weeks of age. The mice were sacrificed at 15 weeks of age. H&E stains of the salivary gland sections from control Ig treatment and B7-H4Ig treatment mice. Arrows indicate infiltrates within the salivary gland (a). Histopathological assessment data are presented as FS (b). (c–e) Serum samples were collected from IgG isotype ctrl treatment and B7-H4Ig treatment NOD/Ltj mice, and the levels of immunoglobulins were measured by ELISA. Immunoglobulin levels for (c) IgM, (d) IgG, and (e) IgA are shown. (f–l) The salivary glands were collected from control Ig treatment and B7-H4Ig treatment NOD/Ltj mice. Relative levels of (f) IL-12 mRNA, (g) IL-6 mRNA, (h) IL-18 mRNA, (i) IL-1*α* mRNA, (j) TNF-*α* mRNA, (k) IFN-*α* mRNA, and (l) BAFF mRNA were determined by quantitative RT-PCR. Two-tailed Student's *t*-test. All the data presented were from three independent experiments.

To evaluate the potential of modulation of the B7-H4 pathway as a therapeutic option to restrict immune-mediated damage, we treated female NOD/Ltj mice with B7-H4Ig and control Ig starting from 8 weeks of age. NOD/Ltj mice were administered with B7-H4Ig (7.5 mg/kg) or control mouse IgG (7.5 mg/kg) every 2 days for 2 weeks. After 7 weeks later, pathological changes of salivary glands with H&E staining were examined. Administration with B7-H4Ig significantly decreased lymphocyte infiltration in salivary glands (Figures 6(a) and 6(b)). B7-H4Ig treatment mice displayed low levels of total IgM (Figure 6(c)) and IgG (Figure 6(d)) in serum, while IgA (Figure 6(e)) was at similar levels in both B7-H4Ig treatment and control Ig treatment. Analysis of inflammatory cytokines in salivary glands after B7-H4Ig treatment revealed that the mRNA levels of IL-12 (Figure 6(f)), IL-6(Figure 6(g)), IL-18 (Figure 6(h)), IL-1*α* (Figure 6(i)), TNF-*α* (Figure 6(j)), and IFN-*α* (Figure 6(k)) were significantly downregulated in B7-H4Ig-treated mice compared to control Ig treatment. The level of BAFF mRNA (Figure 6(l)) in salivary glands did not show any significant differences between the two groups.

### 3.7. B7-H4Ig Ameliorates pSS through Expanding Tregs

**Figure 7:**
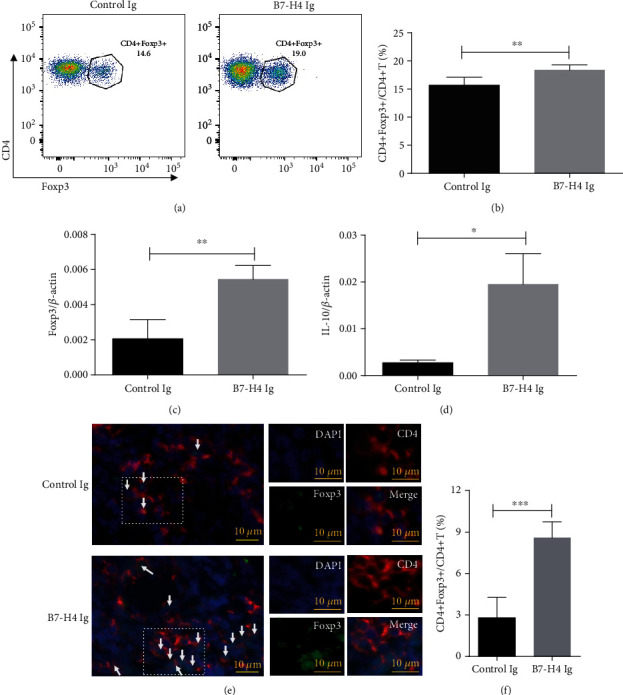
B7-H4Ig ameliorates pSS through expanding Tregs. (a, b) Splenocytes from 15-week-old control Ig treatment and B7-H4Ig treatment NOD/Ltj mice. Staining of (a) CD4 and intracellular staining of Foxp3 cells were performed. (b) Accumulated data of CD4+Foxp3+/CD4+ T cells. (c, d) The salivary glands were collected from control Ig treatment and B7-H4Ig treatment NOD/Ltj mice. Relative levels of (c) Foxp3 mRNA and (d) IL-10 mRNA were determined by quantitative RT-PCR. (e) Colocalization of Foxp3 with CD4 in the salivary glands from B7-H4Ig or control Ig treatment in NOD/Ltj mouse. Anti-Foxp3 (green); anti-CD4 (red); and DAPI (blue). Representative results are shown from three independent experiments (200x magnification). Examples of CD4+Fopx3+ T cells (arrow) are indicated in the left panels. The right panels represent the enlarged portion of the indicated (white hatched box) area in the left panels. (f) Ratio of CD4+Foxp3+/CD4+ T cells in the salivary glands from control Ig or B7-H4 Ig treatment in NOD/Ltj mouse. Two-tailed Student's *t*-test. All the data presented were from three independent experiments.

B7-H4Ig-treated mice had significantly higher levels of CD4+Foxp3+/CD4+ T cells in spleen by flow cytometry analysis than that in those treated with control Ig (Figures 7(a) and 7(b)). In salivary glands, Foxp3 mRNA (Figure 7(c)), together with IL-10 mRNA (Figure 7(d)) level increased in B7-H4Ig-treated mice compared to control Ig-treated mice. Using immunohistochemical analysis, we found that CD4+Foxp3+ T cells increased significantly in B7-H4Ig treatment mice compared to control Ig treatment (Figure 7(e)), and the ratio of CD4+Foxp3+/CD4+ T cells in salivary gland was shown (Figure 7(f)).

## 4. Discussion

pSS is an autoimmune disorder with infiltration of periductal lymphocytes in salivary and lacrimal glands, and it can result in downregulated secretory function, dry mouth, and dry eyes. Due to the extensive involvement of various epithelial cells, pSS has also been depicted as autoimmune epithelitis disease [23]. SGECs can present antigen and induce T cell activation in pSS immunological salivary gland lesions [24]. It was reported that abnormal activation of CD4+ T cells and B cells has close relationship with development and pathogenesis of pSS [25].

B7 costimulatory molecules play an important role in immune-regulatory networks. Previous research shows that B7-2 has been found to be expressed by human nonstimulated monocytes, B cells, and SGECs [26] and negatively regulated by activation processes to costimulate T cell proliferation [27]. B7-H4 (also known as B7S1, B7×, and Vtcn1) is another B7 family member; it is detected on most nonhematopoietic tissues but protein expression is more limited [28]. The biological activity of B7-H4 has been shown to suppress immune responses in autoimmune disease. Soluble B7-H4 can also be detected in the serum of rheumatoid arthritis patients. In mice model, soluble B7-H4 correlates with increased age and disease severity in lupus-prone BWF1 mice and mice with CIA [29]. In an EAE model, administration of B7-H4 antibodies to block B7-H4 during the T cell priming phase led to more severe of EAE [30]. Similarly, EAE was severe in B7-H4 deficient compared to that in WT mice, resulting in the expansion of Th1 and Th17 cells leading to severe disease [30]. The expression of B7-H4 is also induced rapidly in the tubular cells after stimulation with LPS in an autoimmune kidney disease mouse model [30]. In an autoimmune kidney disease mouse model, expression of B7-H4 is rapidly induced in the tubular cells after stimulation with LPS [31]. B7-H4 knockout mice have an enhanced humoral immune response and develop severe renal injury after administration of antibodies against glomerular antigens. It was also observed that B7-H4 deficiency or treatment with soluble B7-H4 could increase the disease incidence and severity in the CIA model [32].

A previous research showed a noticeable decrease in the expression of B7-H4 within the pancreatic islets at approximately 10 weeks of age but a significant loss of B7-H4 expression at 15 weeks in NOD/Ltj mouse [33]. We found the similar changes of B7-H4 expression in salivary glands of NOD/Ltj mice. B7-H4 expression was remarkably reduced in salivary glands of NOD/Ltj mice at 15 weeks compared with that in salivary glands of NOD/Ltj mice at 8 weeks. This result is consistent with the previously published study by Yu et al. [34], suggesting that B7-H4 in SGEC has a potential role in the progression of pSS. Previous studies indicate the remarkable reduction of Treg numbers in both salivary glands and peripheral blood of patients with pSS [35, 36]. It has demonstrated that Tregs play a pivotal role in immune homeostasis by suppressing the proliferation and function of effector T lymphocytes, as well as other immunocytes [37–39]. Whether Tregs have a possible role in the pathogenesis of salivary gland destruction in pSS is not clear. In NOD/Ltj mouse model, we found that Tregs decrease in salivary gland of mice after anti-B7-H4 mAb treatment. Furthermore, blockade B7-H4 in NOD/Ltj mice aggravates the lymphocyte infiltration in salivary glands, increases the serum IgG and IgM levels, and also upregulates the major proinflammatory cytokine production, including IL-12, IL-18, IL-1*α*, TNF-*α*, IFN-*α*, and BAFF in salivary glands. However, there is no significant difference of IL-6 levels between anti-B7-H4 mAb and IgG isotype control treatment. To reveal the relationship between B7-H4 expression and the number/percentage of Treg cells, anti-B7-H4 mAb was found to inhibit the development of Treg cells. It has been reported that cytokines such as IL-4 and TGF-*β* are the factors necessary for Treg differentiation. In anti-B7-H4 mAb-treated mice, IL-4 and TGF-*β* secretions were reduced, suggesting that the changed cytokine expression profile might affect the differentiation of Treg cells. Our study also supports the published mechanism in which the role of B7-H4 suppresses immune responses by inhibition of CD4+ T cell activation [40]. Saliva flow rate is an important clinical index in the diagnosis of pSS. We did not detect saliva flow rate in this research for the following reasons. First, previous research showed secretory dysfunction by 16 weeks of age in NOD/Ltj mouse [41], while in our research, 15-week-old mouse was selected. Second, it has been reported that the presence or extensiveness of lymphocytic infiltration in the salivary or lacrimal glands (sialadenitis or dacryoadenitis, respectively) is not always indicative of degree of secretory dysfunction [42] which indicating that other potential factors may lead to dry mouth even in the absence of lymphocytic infiltration in the salivary glands [43, 44]. Third, this study focused on the relationship between B7-H4 and lymphocytes in NOD/Ltj mouse, and lymphatic infiltration in mouse submandibular gland suggested the preventive effect of B7-H4 in pSS. Finally, in our previous study, there was no correlation between the percentage of B7-H4 expression in the labial gland of pSS patients and clinical indicators (including saliva flow) [34]. Even so, there is no denying that saliva flow rate is an important indicator in Sjogren's syndrome research.

B7-H4Ig is a soluble fusion Fc fusion of the extracellular domain of B7-H4 which has the natural functions of B7-H4 and is able to interact with other immune cells. B7-H4Ig treatment during EAE has shown to alleviate disease through increasing the number and function of Treg cells. Treatment with B7-H4Ig can also reduce the incidence of the autoimmune diabetes in NOD/Ltj mice, as well as the incidence and severity of disease in a CIA model [14, 32]. A phase I study of AMP-110 (a B7-H4Ig fusion protein) for use in patients with RA is ongoing (NTC01878123).

## 5. Conclusions

Our study demonstrated that B7-H4Ig treatment could decrease lymphocyte infiltration in salivary glands of NOD/Ltj mice through expanding Tregs. In the current study, B7-H4 expression in salivary glands was significantly reduced in the model of pSS. Targeting B7-H4 by antibody inhibits Treg differentiation and accelerates pSS, suggesting an important role of B7-H4 in the pathogenesis of pSS. B7-H4Ig ameliorates pSS and promotes Tregs expansion, indicating a new therapeutic approach for clinical treatment of pSS in the future.

## Data Availability

The data used to support the findings of this study are included within the article.
